# Supplementary figures and images for: Increased Heme Oxygenase 1 Expression upon a Primary Exposure to the Respiratory Syncytial Virus and a Secondary Mycobacterium bovis Infection
Source: Antioxidants (Basel). 2022 Jul 26;11(8):1453. doi: 10.3390/antiox11081453 (PMC9332618; doi:10.3390/antiox11081453)

Suppl. Figure S1.

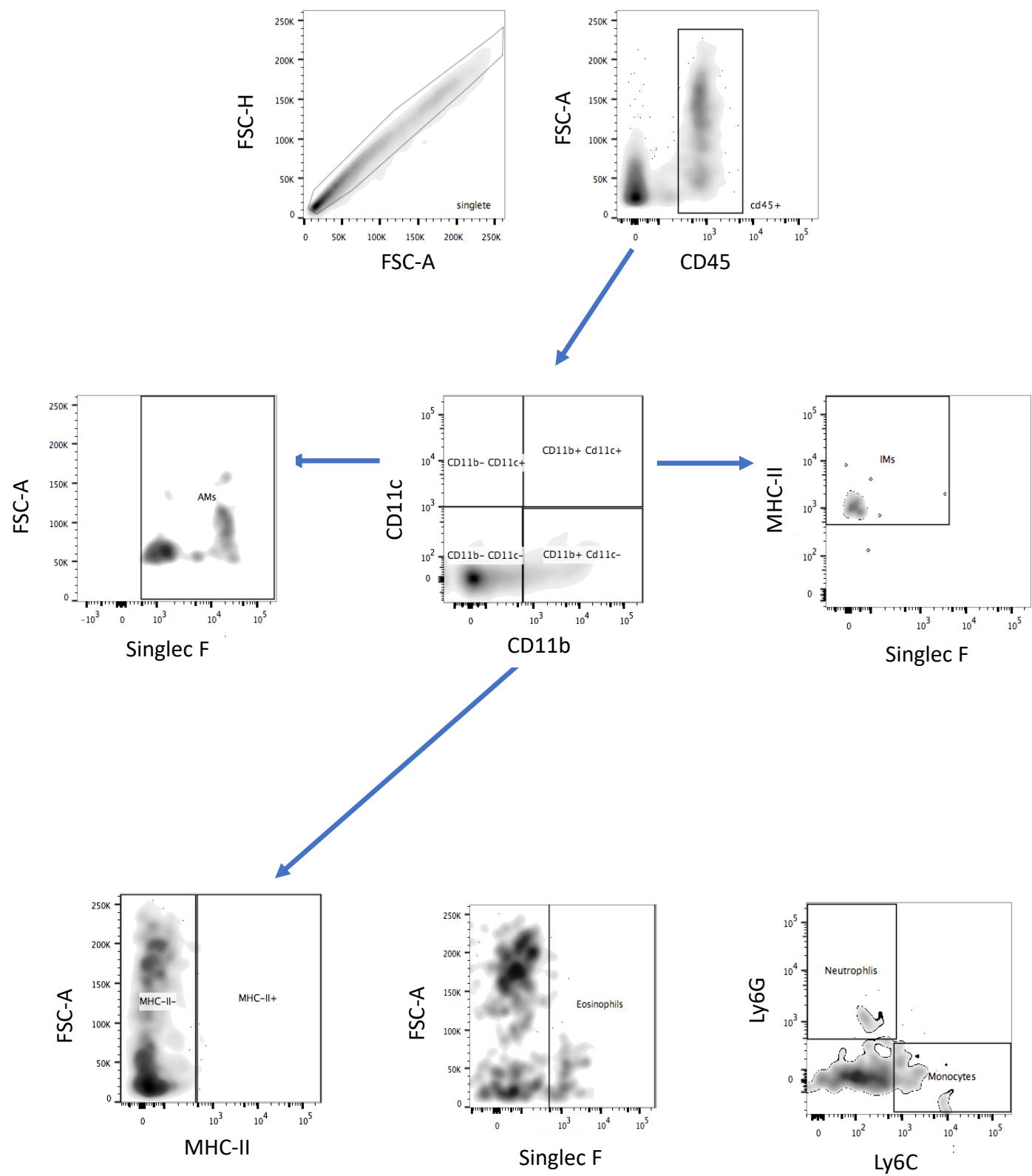

Suppl. Figure S2.

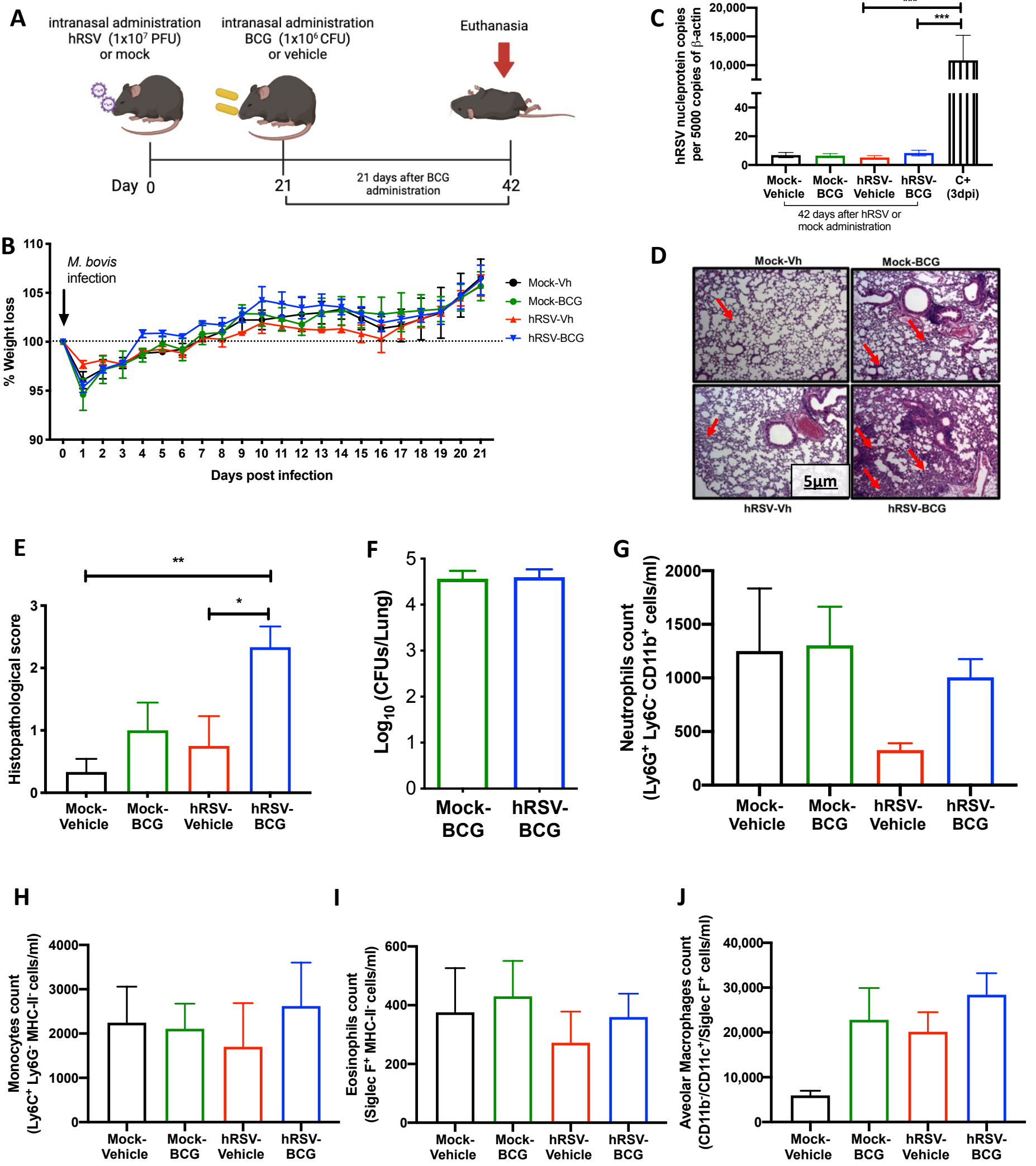

Suppl. Figure S3.

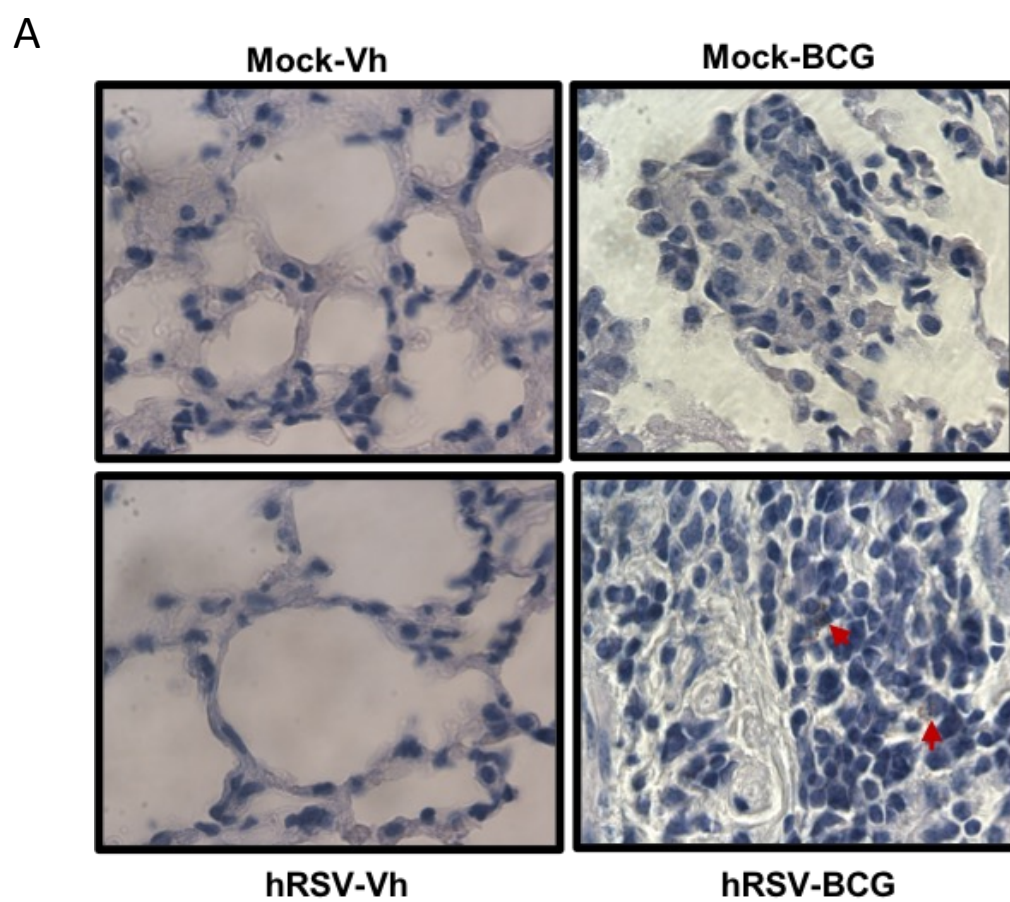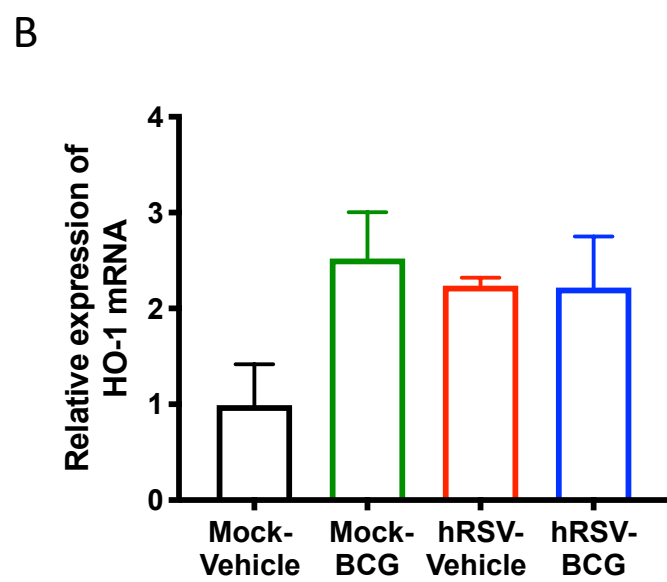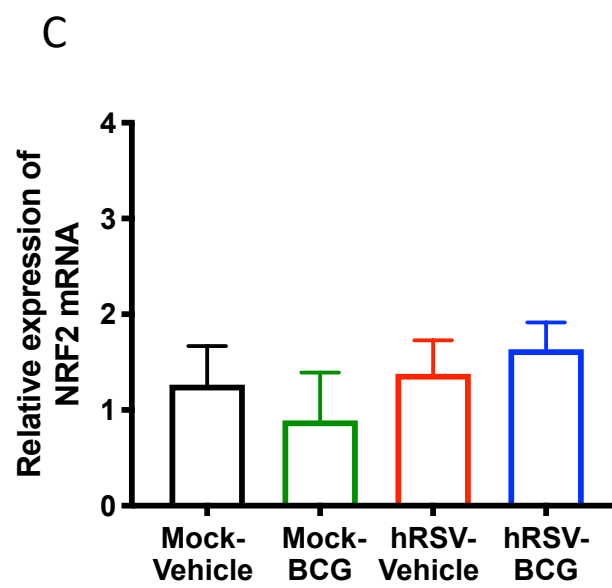

Suppl. Figure S4.

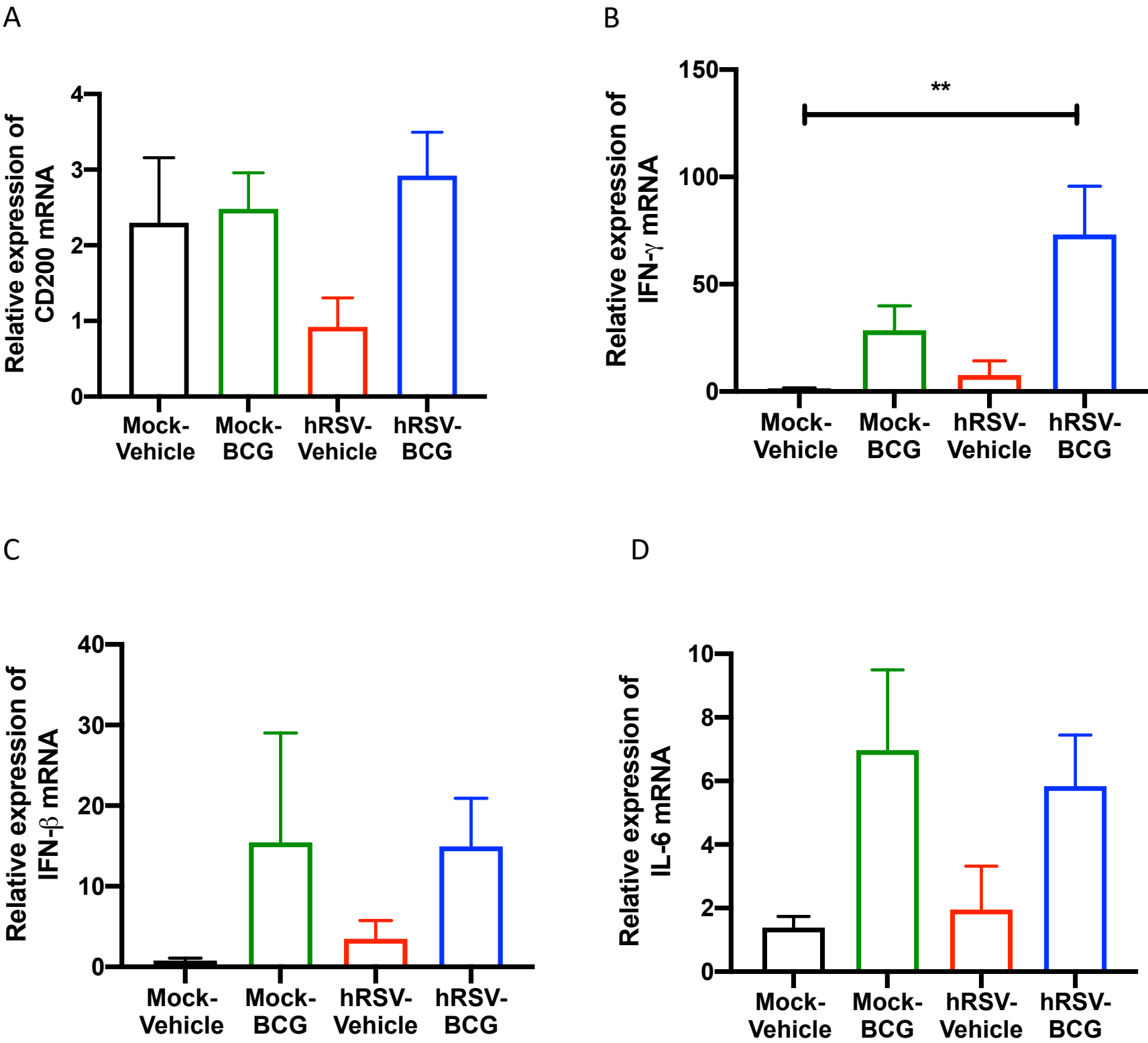

Supplement: Supplementary file 1 [file antioxidants-11-01453-s001.zip › antioxidants-1752963-supplementary.pdf]
